# Integrating metabolite and transcriptome analysis revealed the different mechanisms of characteristic compound biosynthesis and transcriptional regulation in tea flowers

**DOI:** 10.3389/fpls.2022.1016692

**Published:** 2022-09-29

**Authors:** Dingkun Tang, Yihua Shen, Fangdong Li, Rui Yue, Jianwei Duan, Zhili Ye, Ying Lin, Wei Zhou, Yilin Yang, Lixiao Chen, Hongyan Wang, Jian Zhao, Penghui Li

**Affiliations:** ^1^ State Key Laboratory of Tea Plant Biology and Utilization, College of Tea and Food Science and Technology, Anhui Agricultural University, Hefei, China; ^2^ College of Science, Anhui Agricultural University, Hefei, China; ^3^ Municipal Research Institute for Processing of Agricultural and Featured Products, Shiyan Academy of Agricultural Science, Shiyan, China

**Keywords:** tea flowers, transcriptome, metabolism, transcription factor, regulation mechanism, regulation mechanism

## Abstract

The flowers of tea plants (*Camellia sinensis*), as well as tea leaves, contain abundant secondary metabolites and are big potential resources for the extraction of bioactive compounds or preparation of functional foods. However, little is known about the biosynthesis and transcriptional regulation mechanisms of those metabolites in tea flowers, such as terpenoid, flavonol, catechins, caffeine, and theanine. This study finely integrated target and nontarget metabolism analyses to explore the metabolic feature of developing tea flowers. Tea flowers accumulated more abundant terpenoid compounds than young leaves. The transcriptome data of developing flowers and leaves showed that a higher expression level of later genes of terpenoid biosynthesis pathway, such as *Terpene synthases* gene family, in tea flowers was the candidate reason of the more abundant terpenoid compounds than in tea leaves. Differently, even though flavonol and catechin profiling between tea flowers and leaves was similar, the gene family members of flavonoid biosynthesis were selectively expressed by tea flowers and tea leaves. Transcriptome and phylogenetic analyses indicated that the regulatory mechanism of flavonol biosynthesis was perhaps different between tea flowers and leaves. However, the regulatory mechanism of catechin biosynthesis was perhaps similar between tea flowers and leaves. This study not only provides a global vision of metabolism and transcriptome in tea flowers but also uncovered the different mechanisms of biosynthesis and transcriptional regulation of those important compounds.

## Introduction

Tea products, one of the most consumed nonalcoholic beverages, have been popular all over the world due to its pleasant flavors and numerous health benefits (Gilbert, 2019; Zhao et al., 2020). Mostly, the tender shoot tips, including apical buds and young leaves, are used for making various teas, in which many bioactive metabolites are synthesized and accumulated, including catechins, flavonols, caffeine, theanine, and terpenoid volatiles and saponins (Zhao et al., 2020). Tea processing is strictly seasonal—for example, the high-quality green tea was made only in 1 to 2 months of 1 year. Thus, how to comprehensively use the whole tea plant is a key way to improve the income of tea farmers.

For the self-incompatibility of tea plants, the fruit-setting rate is low under natural conditions, but the flowering rate of tea plants is extremely high. In China, over 4.0 billion kilograms of fresh tea flowers were available annually (Chen et al., 2018). More recent evidence have suggested that tea flowers also contained abundant metabolites with high economic and healthy value, and the tea flower was a big potential resource for the extraction of bioactive compounds or preparation of functional foods. However, for a long time, the tea flowers were considered as a “waste” and removed to avoid competing with the tea leaves for nutrients (Chen et al., 2020a). Some chemicals, such as ethephon and α-naphthalene acetic acid, were used to suppress tea plant blossoming to improve the tea yield of tea leaves in the next year (Lin et al., 2003).

The tea flowering time is approximately during August to October in China, and the tea flowers are usually white. Interestingly, pigmented flowers with purple or pink color were found in some mutation cultivars (Zhou, C. et al., 2020; Mei et al., 2021). Purple or pink tea flowers contained abundant anthocyanins compared with white tea flowers. In the pigmented flowers, anthocyanin substances, including delphinidin-3-*O*-glucoside, cyanidin *O*-syringic acid, petunidin 3-*O*-glucoside, and pelargonidin 3-*O*-β-d-glucoside, have been identified (Zhou, C. et al., 2020; Mei et al., 2021). In purple tea flowers, the contents of many volatiles, including benzyl aldehyde, benzyl alcohol, acetophenone, 1-phenylethanol, and 2-phenylethanol, were also higher than those in white tea flowers (Mei et al., 2021). Saponins, which belong to triterpene or steroid glycosides, play crucial roles in terms of health benefits, inducing autophagy and apoptosis in ovarian cancer cells, and inhibiting cancer cell proliferation (Ghosh et al., 2006; Zhao et al., 2015; Fan et al., 2019; Wang et al., 2020). The oleanane-type triterpene saponins were the major type of saponins in tea leaves, seeds, and flowers (Cheok et al., 2014; Cui et al., 2018; Guo et al., 2018). The contents of total saponins were higher in tea flowers than those in young leaves (Chen et al., 2022). At least 24 different saponins were identified in tea flowers, and the key biosynthetic genes and related transcription factors were also predicted based on genome information and RNA-seq data (Fan et al., 2019; Chen et al., 2022). It has been reported that polysaccharide is one of the major bioactive components in tea (Chen et al., 2019). Tea flowers were widely recognized as the main sources of tea polysaccharides, and the content of total saccharides in tea flowers was much higher than that in leaves (Xiao & Jiang, 2015). The polysaccharide from tea flowers has attracted increasing attention among researchers in recent years due to their multiple bioactivities and health benefits, such as anti-oxidant, anti-inflammatory, anti-diabetic, anti-cancer, anti-radiation, anti-obesity, and immunostimulating activities (Chen et al., 2016; Chen et al., 2020b). To date, various polysaccharides have been isolated from tea flowers, the structural properties of which basically consisted of rhamnose, arabinose, xylose, mannose, galactose, galacturonic acid, and glucuronic acid in the monosaccharide composition (Chen et al., 2020a). Moreover, mineral and metabolic analyses also showed that tea flowers, as well as tea leaves, contained not only many mineral elements but also higher primary metabolites, such as sugars, organic acids, and amino acids compared with tea leaves (Jia et al., 2016).

Flavonoid, caffeine, and theanine are the most important characteristic compounds contributing to tea flavor and health benefits (Zhao et al., 2020). Tea flowers and young leaves shared similar types of phenolic compounds; however, tea flowers contained much more hydrolyzed tannins than tea leaves (Shi et al., 2021). In tea flowers, as well as young tea leaves (Shi et al., 2021), epigallocatechin gallate (EGCG) and epicatechin gallate (ECG) were also the major types of catechin. Abundant caffeine was also detected in tea flowers, playing critical roles in the interaction between tea flowers and bees (Huang et al., 2016; Gong et al., 2021). Caffeine has been identified as a kind of nervous system stimulant to improve the brain memory behavior of bees (Wright et al., 2013; Gong et al., 2021; Hempel de Ibarra & Rands, 2021). In tea flowers, many kinds of amino acids, particularly theanine, were also detected (Jia et al., 2016). Theanine, a non-protein amino acid, is also the most abundant amino acid in tea flowers, which is similar with tea leaves (Jia et al., 2016). However, the physiological functions of theanine in tea flowers are largely unknown. Tea flowers also contained and released abundant volatiles with a strong jasmine scent. Liu et al. compared the volatile compounds in tea flowers and leaves and found that the volatiles were mostly derived from the terpenoid biosynthesis pathway, such as monoterpenes and sesquiterpenes (Liu et al., 2017). Zhou et al. also found some tea flower-specific volatiles, such as 1-phenylethanol which was derived from L-phenylalanine (Zhou et al., 2019). Those volatiles play important roles in the interaction between tea flowers and the environment (Chen et al., 2018). Previous studies mostly focused on the volatile compound biosynthesis in tea leaves, but little is known about the mechanism of volatile biosynthesis in tea flowers. Thus, even though the chemical composition of tea flowers is similar to that of young tea leaves, little is known about the biosynthesis and transcriptional regulatory mechanisms of those bioactive compounds in tea flowers.

An integrated approach of metabolite profiling and transcriptome analysis was applied to fully understand the volatile and nonvolatile metabolism and the transcriptional regulation mechanism of those biosynthesis in tea flowers. By target and nontarget metabolic analysis, we comprehensively compared the metabolic characteristics of developing tea flowers with that of young tea leaves and found abundant flower-specific metabolites in tea flowers to highlight the potential function in food processing. Transcriptome analysis uncovered the different mechanism of terpenoid, flavonol, catechins, caffeine, and theanine biosynthesis and transcriptional regulation between tea flowers and leaves. The study provides new insights into the metabolic feature and related biosynthetic mechanism in tea flowers and provides a valuable metabolic and theoretical basis for the potential application of tea flowers in functional food processing.

## Materials and methods

### Plant materials and growth conditions

*C. sinensis* L. cv. SHUCHAZAO, grown at Anhui tea plantation (Anhui Agricultural University), was harvested at the autumn season of 2021 for transcriptome and metabolomic analyses and flower tea processing. The fresh young leaves and flowers of the SHUCHAZAO variety were isolated from the same tree and immediately put into liquid nitrogen for transcriptome and metabolomic analyses. The fresh flowers were divided into six developmental stages, including stage 1 (F1), stage 2 (F2), stage 3 (F3), stage 4 (F4), stage 5 (F5), and stage 6 (F6). The fresh flowers (stage 5) were divided into three parts, namely: receptacle, petal, and stamen. The large-leaf yellow tea samples were purchased from Daheng Yellow Tea Company. The tea-flower tea was made using opening tea flowers according to the standard manufacturing processes of white tea, including collecting the raw material, withering, and drying. The tea and flower samples were subjected to sensory evaluation by a panel of six assessors (three male and three female, 20–45 years old) according to a previous study (Shen et al., 2022).

### Analysis of volatiles by headspace solid-phase microextraction GC-MS

The analysis of volatiles was performed using a previous method by Li et al. (Li et al., 2020). About 0.2 g of tea leaves or flower samples was mixed with 5 ml of boiling water in sealed headspace vials and then kept in a water bath kettle at 50°C. After being in equilibrium for 10 min, an SPME fiber was exposed to the sample headspace for 40 min. The volatile compounds were desorbed at the GC-MS injector for 5 min at 230°C. Then, the analysis process of GCMS-QP2010S was performed to detect the compounds as described by Feng et al. (2019). All reference compounds used in this study were well described by Feng et al. (2019). Ethyl caprate (0.2 μg/g, Sigma, USA) was used as the internal standard to normalize the contents of the compounds.

### Targeted metabolite profiling

The contents of catechins, flavonols, and caffeine were measured by high-performance liquid chromatography (HPLC) according to a method described previously (Li et al., 2022a). Briefly, about 0.05 g of plant tissues was powdered and then mixed with 1 ml 80% methanol extraction solution. The tissues were sonicated at room temperature for 1 h and then shaken overnight at 100 rpm. Following centrifugation at 13,680 *g* for 10 min, the supernatants were filtrated using a Nylon membrane (0.45 μm). The catechins and caffeine were measured by HPLC at 278 nm, and the flavonols were measured by HPLC at 340 nm. Target components were identified by comparing the retention time to authentic compounds. Standard curves were calculated using HPLC and puerarin to quantify the compounds. All standard compounds were purchased from Sigma (USA) and Sangon Biotech (Shanghai, China).

### Non-targeted metabolomics

The non-targeted metabolomics method was performed by Waters Acquity I-Class PLUS ultra-high performance liquid tandem Waters Xevo G2-XS QTOF high-resolution mass spectrometer with a Waters Acquity UPLC HSS T3 column (1.8 um, 2.1*100 mm). Six repetitions of each sample were carried out. The positive and negative ion modes were used with 1-μl injection volume. A binary gradient elution system was adopted, and the mobile phase consisted of 0.1% formic acid aqueous solution (A) and 0.1% formic acid acetonitrile (B). The flow rate was maintained at 0.4 ml/min. The LC-MS/MS data were collected by the acquisition software MassLynx V4.2. The low collision energy is 2 V, and the high collision energy range is 10–40 V. The scanning frequency is 0.2 s for a mass spectrum. The parameters of the ESI ion source are as follows: capillary voltage, 2,000 V (positive ion mode) or -1,500 V (negative ion mode); cone voltage, 30 V; ion source temperature, 150°C; desolvent gas temperature, 500°C; backflush gas flow rate, 50 L/h; and desolventizing gas flow rate, 800 L/h. The raw data collected using MassLynx V4.2 is processed by Progenesis QI software for peak extraction, peak alignment, and other data processing operations based on the Progenesis QI software online METLIN database and Biomark’s self-built library for identification. The metabolites were identified by searching the internal database and public databases (KNApSAcK, HMDB, MoTo DB, and METLIN). For the internal database, it was constructed based on the standard materials and purified compounds. Additionally, some public databases (MassBank, KNApSAcK, HMDB, and METLIN) also contain some information of metabolites that can be referenced directly. The metabolites were identified by comparing the accurate precursor ion (Q1) and production (Q3) values, retention time, and fragmentation pattern with the database. After normalizing the original peak area information with the total peak area, a follow-up analysis was performed. Principal component analysis and Spearman correlation analysis were used to judge the repeatability of the samples within group and the quality control samples. The identified compounds are searched for classification and pathway information in Kyoto Encyclopedia of Genes and Genomes (KEGG), HMDB, and lipidmaps databases. According to the grouping information, we calculated and compared the difference multiples. *T*-test was used to calculate the significant difference (*P*-value) of each compound. The R language package ropls was used to perform OPLS-DA modeling (Thévenot et al., 2015), and 200 times of permutation tests were performed to verify the reliability of the model. The variable importance in projection (VIP) value of the model was calculated using multiple cross-validation. The method of combining the difference multiple, the *P*-value, and the VIP value of the OPLS-DA model was adopted to screen the differential metabolites. The screening criteria are *P*-value (fold change) <0.05 and VIP >1. The significance of the different metabolites of the KEGG pathway enrichment was calculated using the hypergeometric distribution test.

### RNA extension and transcriptome analysis

For the RNA-Seq analysis, total RNA was isolated from tea flowers with Trizol reagent (Invitrogen) and was purified, and its quality and concentration were evaluated by using Agilent 2100 Bioanalyzer. cDNA library construction and RNA-Seq were performed as previously described on an Illumina HiSeq2500 instrument (Li et al., 2022b). The clean data were mapped to the tea plant genome (Wei et al., 2018) by using TopHat2 software (Kim et al., 2013). Transcripts per million (TPM) were introduced to qualify the expression levels of transcripts.

### Bioinformatic and statistical analysis

The pHeatmap R package (https://www.rdocumentation.org/packages/pheatmap) and Tbtools (Chen et al., 2020) were used to structure the heat map of gene expressions. A phylogenetic tree of MYB proteins was built by MEGA7.0 software using the neighbor-joining method with 1,000 bootstrap replications (Kumar et al., 2016). *corrplot* in the R package was used for the correlation analysis of gene expression patterns and drawing diagrams (Li et al., 2022a).

### Data availability

All transcriptome raw data generated in this study have been deposited in NCBI Sequence Read Archive BioProject number PRJNA798825 (https://www.ncbi.nlm.nih.gov/sra, SRR21524583-SRR21524600), and the metabolic data are listed in **Supplementary Table S1**.

## Results

### New application of tea flowers in tea processing

**Figure 1:**
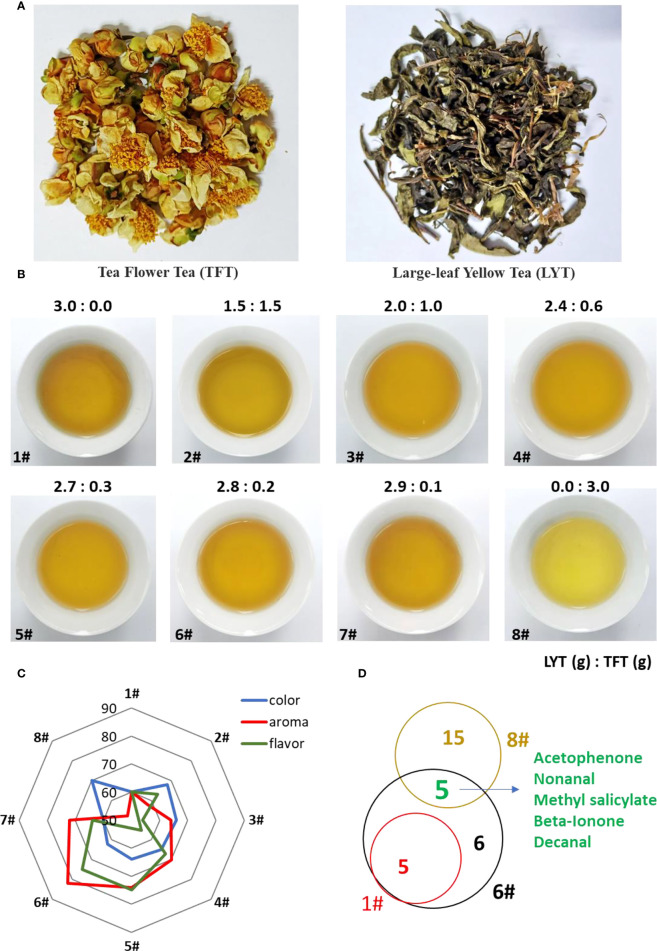
Potential application of tea flowers in tea processing. **(A)** Appearance of tea flower tea products. TFT, tea flower tea; LYT, large-leaf yellow tea. **(B)** Soup color from mixed tea products with different ratios between TFT and LYT. The ratio number between TFT and LYT is listed on top of each figure. **(C)** Effect of TFT on taste attributes. **(D)** Venn diagram showing the volatile compounds of tea flowers contributing to tea aroma.

It is important to research new applications of tea flowers for improving the economic value thereof. Large-leaf yellow tea (LYT) is one kind of yellow-type tea which has multiple health benefits, such as alleviating alcoholic liver disease and inhibiting adipose tissue hypertrophy (Xu et al., 2021; Wang et al., 2022; Wu et al., 2022). However, LYT is made from old tea leaves and stems, leading to poor aroma and flavor or being unpopular. In order to further explore the application of tea flowers, we used dry tea flowers (TFT) to improve the flavor quality of LYT (**Figure 1A**). The sensory evaluation analysis showed that the best ratio of LYT to TFT was 2.8:0.2 (#6 sample), which could significantly improve the flavor quality of LYT (**Figures 1B, C**). The GC-MS analysis also showed that many volatiles derived from flowers were detected in #6 sample, such as nonanal and decanal, which contributed to the tea quality (**Figure 1D**).

### Metabolic analysis of tea flowers

**Figure 2:**
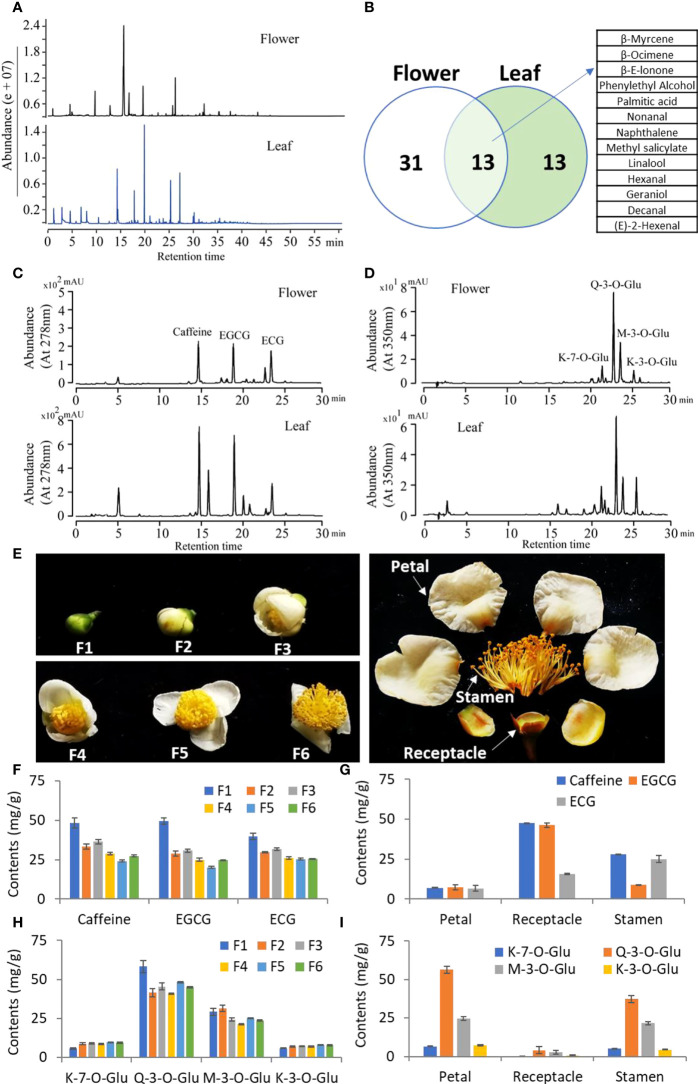
Metabolic analysis of tea flowers and leaves. **(A)** GC-MS total ion current of volatiles in tea flowers and leaves. **(B)** Venn diagram showing the variation of volatile compounds in tea flowers and leaves. **(C, D)** HPLC chromatographs of catechins and caffeine **(C)** and flavonols **(D)** in tea flowers and leaves. The compound names are listed on top of the peaks. EGCG, epigallocatechin gallate; ECG, epicatechin gallate; K-7-0-Glu, kaempferol-7-O-glucoside; K-3-0-Glu, kaempferol-3-O-glucoside; Q-3-0-Glu, quercetin-3-O-glucoside; M-3-0-Glu, myricetin 3-O-glucoside. **(E)** Phenotype of developing tea flowers. F1, flower stage 1; F2, flower stage 2; F3, flower stage 3; F4, flower stage 4; F5, flower stage 5; F6, flower stage 6. **(F, G)** Contents of caffeine and catechins in developing tea flowers **(F)** and different parts of tea flowers **(G)**. **(H, I)** Contents of flavonols in developing tea flowers **(H)** and different parts of tea flowers **(I)**. All data are from at least three biological replicates and are expressed as mean ± SD.

We then compared the profile of volatile compounds in young leaves and opening flowers of tea plants using GC-MS. The compound profiling showed significant differences between leaves and flowers (**Figure 2A**). A total of 44 and 26 compounds were identified in young leaves and flowers, respectively, by comparing standards and the NST library (**Figure 2B**). Between them, 13 compounds, such as linalool, hexanal, and geraniol, were shared in young leaves and flowers, and 31 compounds were highly detected in flowers (**Figure 2B**). Then, we checked the nonvolatile compounds in tea flowers and leaves. The HPLC analysis results showed a similar pattern with low abundance in tea flowers compared with that in leaves (**Figure 2C**). In flowers, EGCG and ECG have the highest content of catechins, which is consistent with the leaves (**Figure 2C**). We also found that caffeine was the predominant purine alkaloid compound in tea flowers as well as in tea leaves (**Figure 2C**). The flavonol compounds were also analyzed, and similar flavonol profiling was also detected between flowers and leaves (**Figure 2D**).

In order to further analyze the dynamic metabolism of tea flowers, the developing tea flowers (F1–F6) and petal, receptacle, and stamen were separately analyzed (**Figure 2E**). We found that the contents of caffeine, EGCG, and ECG showed a dramatic decline during flower development (**Figure 2F**). Otherwise, caffeine was mainly accumulated in the receptacle and stamen, and EGCG only mainly accumulated in the receptacle (**Figure 2G**). However, ECG was highly accumulated in the stamen and in the receptacle next (**Figure 2G**). The flavonol compounds, including quercetin-3-*O*-glucoside (Q-3-*O*-Glu) and myricetin 3-*O*-glucoside (M-3-*O*-Glu), also showed a decline during flower development; however, kaempferol-7-*O*-glucoside (K-7-*O*-Glu) and kaempferol-3-*O*-glucoside (K-3-*O*-Glu) showed a slight elevation during flower development (**Figure 2H**). Interestingly, those flavonol compounds were specially accumulated in petals and stamens, which was distinctly different from the distribution of caffeine or catechins (**Figure 2I**).

### Non-target metabolomic analysis of tea leaves and flowers

**Figure 3:**
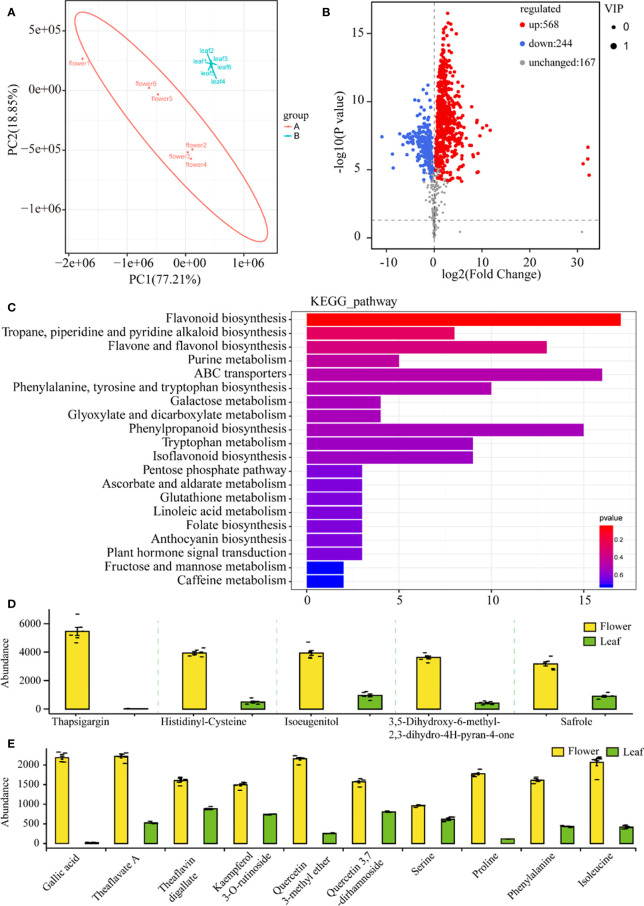
Nontarget metabolic analysis of tea flowers and leaves. **(A)** Principal component analysis of tea flower and leaf samples. PC1, the first principal component. PC2, the second principal component. For each tissue samples, six biological replicates were prepared. **(B)** Volcano plots of differential metabolites in tea flower and leaf samples. **(C)** Kyoto Encyclopedia of Genes and Genomes pathway enrichment analysis of different metabolites in tea flower and leaf samples. **(D)** Five most abundant metabolites in tea flowers. **(E)** Differences in key metabolites between tea flowers and leaves.

Freshly opening flowers and young leaves (first leaf with bud) were harvested at the same time, and nontargeted analysis for flowers and leaves was performed on the UPLC-QTOF/MS platform in positive and negative ion modes. In total, 18,070 metabolite ion features were detected in flowers and leaves, in which 979 metabolites were structurally annotated (**Supplementary Table S1**). After the pretreatment of the metabolite data, an unsupervised PCA was performed to monitor the overview of the difference in the metabolite phenotypes between the tea and flower samples. The first two principal components explained 96.06% of the total variance (77.21 and 18.85%, respectively) (**Figure 3A**). The metabolite phenotypes of the flower samples were remarkably different from those of the leaf samples (**Figure 3A**). To identify differential metabolites between tea flowers and tea leaves, we selected metabolites with a fold change ≥1 (upregulated or downregulated) in flowers compared to leaves. These metabolites were screened using a VIP value (VIP ≥1) from the OPLS-DA model and *P*-value <0.05. In total, 812 differential metabolites were detected between flower and leaf samples. Of these, 568 metabolites were downregulated, and 244 metabolites were upregulated in flowers compared with leaves (**Figure 3B**). Then, the 812 metabolites were mapped to the KEGG database to look first at information about pathways. The KEGG pathway enrichment analysis showed that many metabolites participated in secondary metabolism processing, such as “flavonoid biosynthesis”, “phenylpropanoid biosynthesis”, “flavone and flavonol biosynthesis”, and amino acid metabolism processing, such as “ABC transporters”, and “phenylalanine, tyrosine, and tryptophan biosynthesis” (**Figure 3C**). The five most highly represented metabolites in tea flowers are shown in **Figure 3D**. The contents of those compounds in flowers were significantly higher than that in leaves (**Figure 3D**). Those metabolites were always used for essential oil and spice production and as raw materials for antibacterial drugs. The abundance of those metabolites suggested the potential application of tea flowers in comprehensive processing. Gallic acid and its derivatives play critical roles in hydrolyzable tannins, condensed tannins, and galloylated flavan-3-ols biosynthesis (Wei et al., 2018). Notably, the content of gallic acid in flowers is higher than in leaves by more than 80-fold (**Figure 3E**). However, the contents of galloylated catechins, such as EGCG and ECG, are significantly lower in flowers than that in leaves (**Supplementary Figure S1**). We also found several flavonol compounds, such as kaempferol, kaempferol 3-o-rutinoside, quercetin 3-methyl ether, and quercetin 3,7-dirhamnoside, to be highly accumulated in flowers compared with leaves (**Figure 3E**). Theanine, a non-protein amino acid, is another characteristic secondary metabolite in tea, which is synthesized in tea roots and then transported to leaves for storage (Wei et al., 2018). Abundant theanine was detected in flowers, with a lower concentration than that in leaves (**Supplementary Figure S1** and **Supplementary Table S1**). We also found some amino acids, such as L-serine, L-proline, L-phenylalanine, and L-isoleucine, to be highly accumulated in flowers compared with those in leaves (**Figure 3D**).

### Tea flowers expressed terpenoid biosynthesis genes for diverse volatile production

In order to reveal the mechanism of metabolic difference between leaves and flowers, a transcriptome analysis of tea flowers was performed, including developing flowers (stage 1, F1; stage 2, F2; stage 3, F3; stage 4, F4; stage 5, F5; and stage 6, F6) and receptacle, petal, and stamen. In developing flowers, 3,089 differentially expressed genes were identified (**Supplementary Table S2**). The KEGG analysis showed that many differentially expressed genes participated (presented in **Supplementary Figure S2**), such as “pectinesterase”, “pectate lyase”, “glutathione S-transferase”, and “flavonol synthase”. We also compared the gene expression profile in the receptacle, petal, and stamen. In total, 23,260, 20,379, and 21,026 genes were expressed in the receptacle, petal, and stamen, respectively (TPM > 1) (**Supplementary Figure S3**). The Venn diagram showed that the transcripts of 18,468 genes (73.9%) were all detected in the receptacle, petal, and stamen (**Supplementary Figure S3**). In tea flowers, the abundance of volatiles, particularly the terpenoid compounds, was detected. Then, we analyzed the terpenoid biosynthesis in flowers, and the transcriptome data of developing leaves and stems was used for comparison as control, which was generated by our previous study (Zhang et al., 2022). Most early genes involved in terpenoid biosynthesis showed low expression levels in F1, then increased in F2 and F3, and then kept a high level in opening flowers (**Supplementary Table S3**). We also found the similar expression patterns of those early genes in developing flowers and leaves (**Supplementary Table S3**)—for instance, two *HMG-CoA reductases* (*HMMG-2* and *HMMG-4*) are the predominant genes in both flowers and leaves (**Supplementary Table S3**). The similar expression patterns suggested that the methylerythritol phosphate (MEP) and mevalonate pathways were conservative in flowers and leaves. Wo also found the genes involved in triterpenoid genes, such as *β-amyrin synthase* and *dammarenediol II synthase*, to be highly expressed in the receptacle and stamen compared with the petal or highly expressed in leaves and stems compared with young shoots (**Supplementary Table S3**).

**Figure 4:**
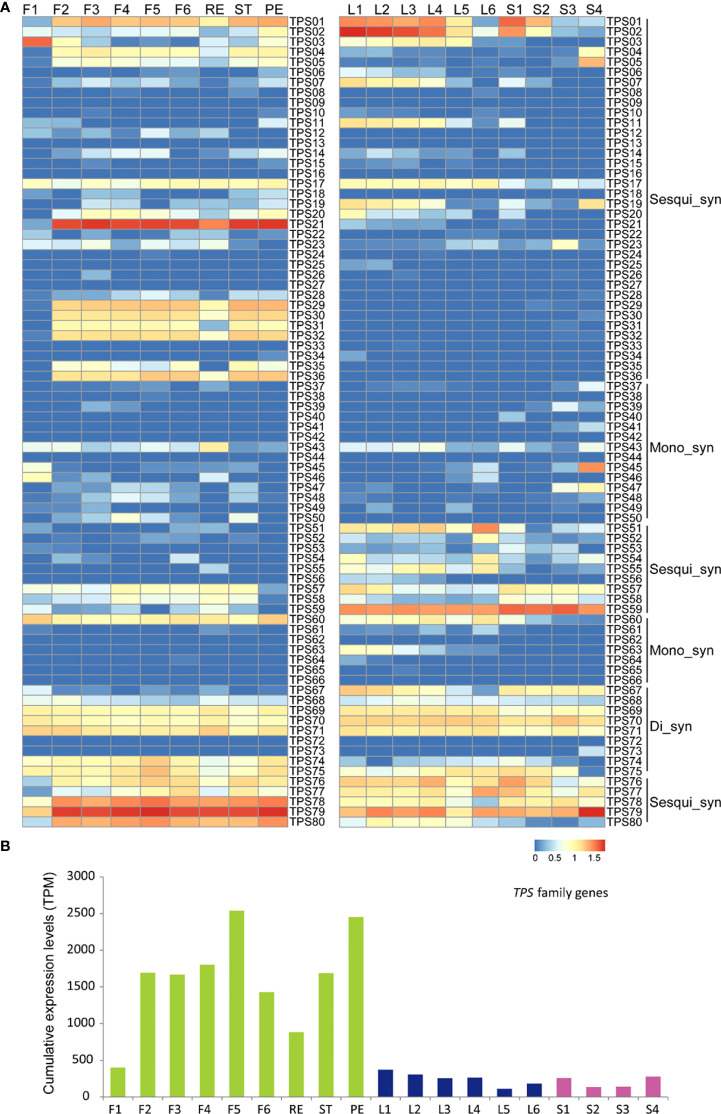
Expression analysis of *TPS* gene family in tea developing flowers and leaves. **(A)** Heat map expression analysis of *TPS* genes involved in the terpenoid process in tea developing flowers and leaves. F1, tea flower stage 1; F2, tea flower stage 2; F3, tea flower stage 3; F4, tea flower stage 4; F5, tea flower stage 5; F6, tea flower stage 6; ST, stamen; PE, petal; RE, receptacle; L1, first tea leaf; L2, second tea leaf; L3, third tea leaf; L4, fourth tea leaf; L5, fifth tea leaf; and L6, sixth tea leaf; S1, internode between the first leaf and second leaf; S2, internode between the second leaf and third leaf; S3, internode between the third leaf and fourth leaf; S4, internode between the fourth leaf and fifth leaf; Mono_syn, monoterpene synthase; Sesqui_syn, sesquiterpene synthase; Di_syn, diterpene synthase. The expression level [log10(FPKM)] of each gene is shown in the heat map boxes. **(B)** Cumulative expression analysis of the *TPS* family genes in tea developing flowers and leaves.

Thus, the divergence of later genes involved in terpenoid biosynthesis, such as *terpene synthase* (*TPS*) genes, is the reason for the significant difference of the terpenoid compound between leaves and flowers. In tea plants, 80 *TPS* members were identified, which were predicted or reported to be involved in monoterpene and sesquiterpene biosynthesis (Zhou, H. C. et al., 2020). The expression profile of the *TPS* gene family in leaves and flowers was compared (**Supplementary Table S4**). Most *TPS* genes also showed a low expression level in F1, and then the expression levels increased in opening flowers (**Figure 4A**). We also noted that most *TPS* genes were highly expressed in petals and stamens, compared with the receptacle, such as *TPS01*, *TPS21*, and *TPS29*. Some *TPS* genes included in the Di_syn clade, such as *TPS69*, *TPS70*, and *TPS71*, showed high expression levels during flower development and in the receptacle, petal, and stamen (**Figure 4A**). In tea leaves, most *TPS* genes included in the Sesqui_syn and Mono_syn clades, such as *TPS01*, *TPS02*, and *TPS07*, are highly expressed in young leaves and young stems (**Figure 4A**). Similar to flowers, the leaves also highly expressed many Di-syn *TPS* genes in all leaf and stem samples (**Figure 4A**). Not only the *TPS* genes showed different expression patterns in developing flowers and leaves but also the flowers and leaves specially expressed different *TPS* members, which may lead to the divergent terpenoid metabolites—for instance, *TPS02*, *TPS11*, *TPS19*, and *TPS51* showed higher expression levels in leaves than in flowers, and on the contrary, *TPS04*, *TPS21*, *TPS29*, *TPS30*, and *TPS31* showed higher expression levels in flowers than in leaves (**Figure 4A**).

In tea plants, some *TPS* genes have been identified in Ses_syn biosynthesis, including the linalool-biosynthesized *TPS76* and *TPS77* and the nerolidol-biosynthesized *TPS79* (Zhou, H. C. et al., 2020). Those genes showed conservatively high expression levels in leaves and flowers (**Figure 4A**), which was consistent with the high concentration of linalool in both leaves and flowers (Liu et al., 2017). The cumulative expression analysis further showed that, in F1, the *TPS* gene family was lowly expressed and then rapidly induced as the flower is developing (**Figure 4B**). In F5 stage, the highest expression levels of *TPS* gene family were detected (**Figure 4B**). Expectedly, the *TPS* gene family was highly expressed in petals, followed by stamens and receptacles (**Figure 4B**). Compared with the flowers, the *TPS* gene family showed a significantly lower expression level in developing leaves and stems, which was consistent with the lower abundance of total terpenoid compounds in leaves and stems (**Figure 4B**).

### Flavonol and catechin biosynthesis in tea flowers

**Figure 5:**
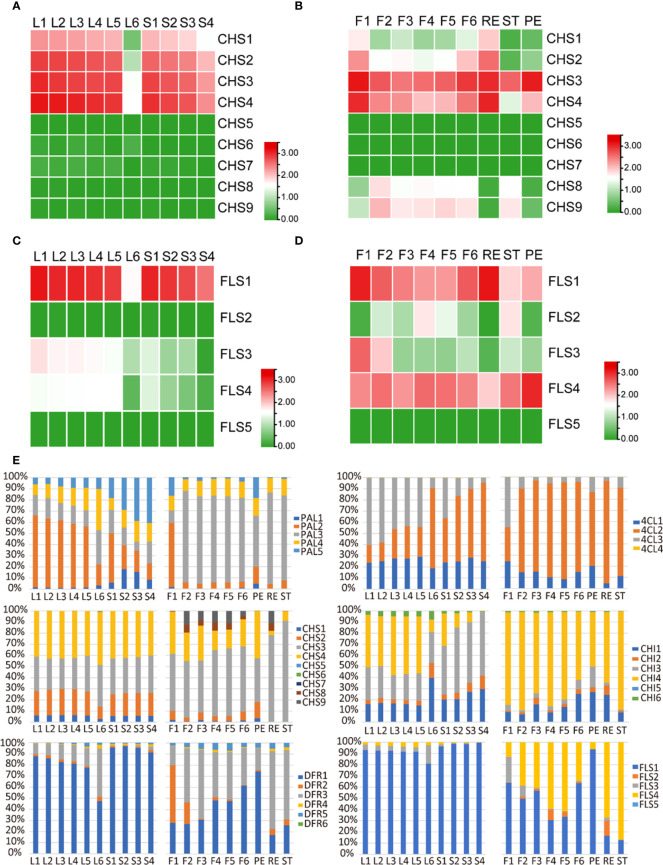
Expression analysis of gene families involved in flavonoid biosynthesis in tea developing flowers and leaves. **(A, B)** Heat map expression analysis of *CHS* genes in tea developing flowers **(A)** and leaves **(B)**. **(C, D)** Heat map expression analysis of *FLS* genes in tea developing flowers **(C)** and leaves **(D)**. **(E)** Relative expression level of gene family members in different tissues of tea plants. F1, tea flower stage 1; F2, tea flower stage 2; F3, tea flower stage 3; F4, tea flower stage 4; F5, tea flower stage 5; F6, tea flower stage 6; ST, stamen; PE, petal; RE, receptacle; L1, first tea leaf; L2, second tea leaf; L3, third tea leaf; L4, fourth tea leaf; L5, fifth tea leaf; L6, sixth tea leaf; S1, internode between the first leaf and second leaf; S2, internode between the second leaf and third leaf; S3, internode between the third leaf and fourth leaf; S4, internode between the fourth leaf and fifth leaf; PAL, phenylalanine ammonialyase; 4CL, 4-coumarate:CoA ligase; CHS, chalcone synthase; CHI, chalcone isomerase; DFR, dihydroflavonol reductase; FLS, flavonol synthase. The expression level [log10(FPKM)] of each gene is shown in the heat map boxes.

Flavonoid compounds, such as flavonols and catechins, play crucial roles in not only tea flavor formation but also multiple stress resistance in tea plants (Zhao et al., 2020). In **Figure 2**, we have analyzed the flavonoid metabolite profile in tea leaves and flowers, which suggested a similar metabolite pattern in different tissues. However, the transcriptome feature of those flavonoid biosynthetic genes in flowers and leaves is unknown. Flavonoid pathway is derived from the phenylpropanoid process. We analyzed the expression of key genes involved in the phenylpropanoid pathway, and those genes showed a different expression pattern between a developing leaf and developing flowers (**Supplementary Figure S4**). Chalcone synthase (CHS) is the first limited enzyme in the flavonoid biosynthesis pathway, which recognized the substrate 4-coumaroyl CoA to compete with the lignin biosynthesis pathway. In the tea genome, nine *CHS* copies were identified (Wei et al., 2018). In order to investigate in detail the function of each *CHS* gene, we compared the *CHS* expression pattern in leaves and flowers. In tea leaves, only four *CHS* members, including *CHS1*, *CH2*, *CHS3*, and *CHS4*, were expressed (**Figure 5A**). The expression of those genes was high in young leaves and stems and then decreased as these developed (**Figure 5A**). In flowers, besides *CHS1*, *CH2*, *CHS3*, and *CHS4*, which were highly expressed in leaves, *CHS8* and *CHS9* were also specially expressed in flowers (**Figure 5B**). *CHS5*, *CHS6*, and *CHS7* were also not expressed in flowers (**Figure 5B**). In addition, *CHS1* and *CHS2* were only expressed in receptacles (RE), and *CHS3* and *CHS4* were expressed in whole flowers (**Figure 5B**). Notably, *CHS8* and *CHS9* were specially expressed in stems (ST) (**Figure 5B**), which suggests the important function in pollen fertility.

Flavonols are a particular class of flavonoids which are highly accumulated in young leaves and stems and play major roles in the bitter taste (Zhao et al., 2021). Interestingly, in tea flowers, flavonols were specially accumulated in ST and PE and not detected in RE (**Figure 2**). Then, we analyzed the expression pattern of *Flavonol synthase* (*FLS*) genes in tea plants. At least five *FLS* gene copies were identified in the tea genome. In leaves, only three of five *FLS* genes, including *FLS1*, *FLS3*, and *FLS4*, were expressed, in which *FLS1* was the major gene with highest expression level, followed by *FLS3* and *FLS4* (**Figure 5C**). In flowers, except *FLS5*, all four other *FLS* genes were expressed, in which *FLS1* and *FLS4* were the major genes with the highest expression levels (**Figure 5D**). *FLS1* was highly expressed in RE, and *FLS4* was highly expressed in PE (**Figure 5D**). We also noticed that *FLS2* was specially expressed in ST, which suggests an important function in pollen fertility just like *CHS8* and *CHS9* (**Figure 5D**).

In addition, we further analyzed the expression patterns of other biosynthetic genes and also found an expression divergence of the gene family in leaves and flowers. Phenylalanine ammonialyase (PAL) is the first enzyme of the phenylpropanoid pathway. In tea leaves, *PAL2*, the major gene with the highest expression levels, was highly expressed in young leaves and then decreased during development (**Figure 5E**). On the contrary, the expression of *PAL3* and *PAL4* showed an elevation during development. However, in developing flowers or different parts of flowers, *PAL3* is always the major gene (**Figure 5E**). *4-Coumarate : CoA ligase* (*4CL3*) was highly expressed in young leaves and then decreased during leaf development. Conversely, the expression level of *4CL2* decreased during leaf development. In tea flowers, only *4CL2* is the major gene (**Figure 5E**). Other members of the gene family, such as *CHS*, *chalcone isomerase*, *dihydroflavonol reductase*, and *FLS*, also showed a different expression priority in leaves and flowers (**Figure 5E**).

### Regulation mechanism of flavonols and catechin biosynthesis in tea flowers

**Figure 6:**
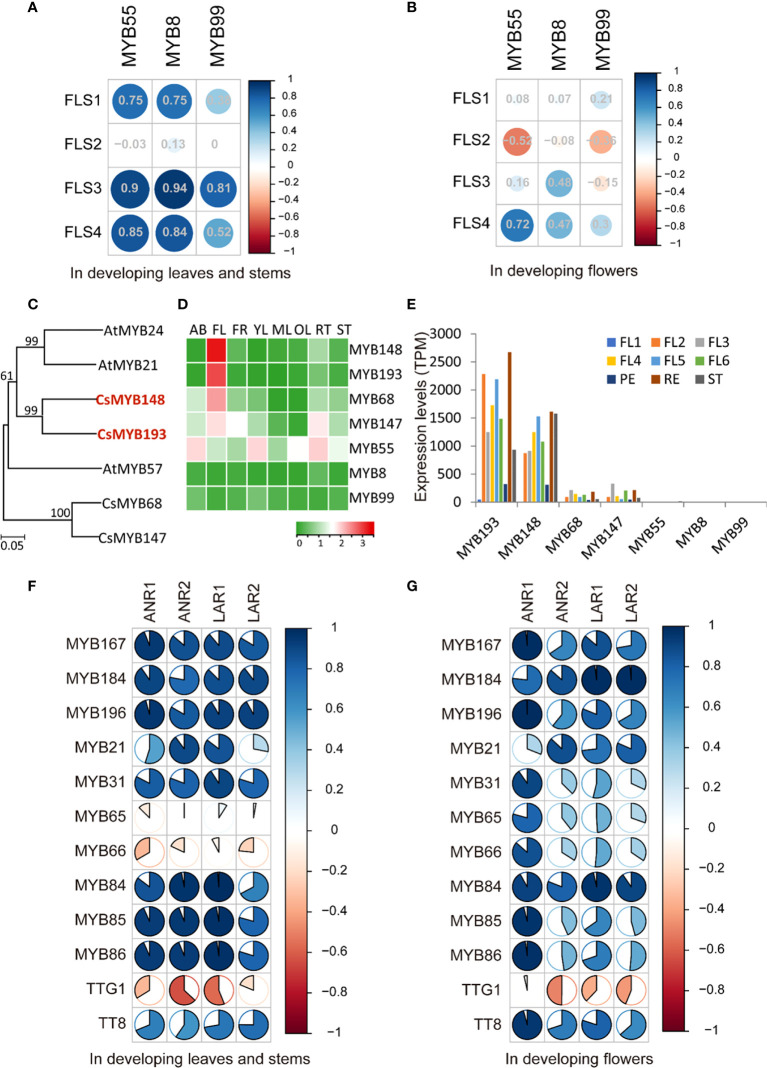
Transcriptional regulatory mechanism of flavonol and catechin biosynthesis in tea flowers. **(A, B)** Pearson correlation analysis of the expression of *FLS* genes and the expression of three flavonol MYB regulator genes in developing leaves **(A)** and developing flowers **(B)**. The sizes of circles indicate the degree of correlation. Green indicates a positive correlation, and red indicates a negative correlation. **(C)** Phylogenetic tree of tea MYBs with others related to flavonol biosynthesis regulation in flowers. The numbers at the nodes indicate the bootstrap value with 1,000 replicates. The red MYB genes highlight their corresponding homolog genes in tea plants. **(D)** Heat map expression analysis of candidate MYB genes in different tea tissues. AB, apical bud; FL, flower; FR, fruit; YL, young leaf; ML, mature leaf; OL, old leaf, RT, root, ST, stem. The expression level [log10(FPKM)] of each gene is shown in the heat map boxes. **(E)** Expression level analysis of candidate MYB genes regulating flavonol biosynthesis in tea developing flowers. **(F, G)** Pearson correlation analysis of the expression of *ANR* and *LAR* genes and the expression of catechin regulator genes in developing leaves **(F)** and developing flowers **(G)**.

In leaves and flowers, a similar flavonoid profile was detected; however, the expression of those biosynthetic gene families showed a dramatic difference in those tissues. Thus, we further analyzed the regulatory mechanism of flavonoid biosynthesis in leaves and flowers. In our previous study, we have comprehensively analyzed the transcription factors regulating flavonoid biosynthesis in tea plants, in which three MYB transcription factors regulate flavonol biosynthesis in leaves, including MYB8, MYB55, and MYB99 (Li et al., 2022b). In developing tea leaves and stems, the expression patterns of those MYB transcription factor genes were highly correlated with the expression of *FLS* family genes (**Figure 6A**). In developing flowers, no correlation between the expression pattern of *MYB* and *FLS* genes was detected based on the transcriptome data (**Figure 6B**). This suggested the different regulatory network of flavonol biosynthesis in leaves and flowers. In *Arabidopsis*, AtMYB21 and AtMYB24 specially regulate flavonol biosynthesis in flowers (Zhang et al., 2021). To explore the regulation mechanism of flavonol biosynthesis in tea flowers, the homologous genes of AtMYB21 and AtMYB24 in tea plants were identified. In the tea plant genome, at least four candidate *MYB* genes, including *MYB148*, *MYB193*, *MYB68*, and *MYB147*, were identified (**Figure 6C** and **Supplementary Figure S5**). Those candidate genes, particularly *MYB148* and *MYB193*, were specially expressed in flowers (**Figure 6D**). However, *MYB55*, *MYB8*, and *MYB99* were not expressed in flowers (**Figure 6D**). In addition, *MYB148* and *MYB193* showed higher expression levels in ST and PE compared with RE (**Figure 6E**), which is consistent with the higher content of flavonol compounds in ST and PE. Those data strongly indicated that *MYB148* and *MYB193* conservatively participated in flavonol biosynthesis regulation in flowers, which is different with the regulatory network in tea leaves. Then, we analyzed the catechin biosynthesis regulation in tea flowers. Our previous study has shown that, in tea plants, MYB-bHLH-WD40 (MBW) complexes play crucial roles in catechin biosynthesis (Li et al., 2022a; Li et al., 2022b). At least 10 MYB transcription factors were confirmed as the component of the MBW complex regulating catechin biosynthesis in tea plants (Li et al., 2022b). We found both in tea leaves and flowers that the expression patterns of those transcription factor genes were highly corelated with the expression of key biosynthetic genes, such as *anthocyanidin reductase* (*ANR*) and *leucoanthocyanidin reductase* (*LAR*), suggesting the conservative regulatory mechanism of catechin biosynthesis in leaves and flowers (**Figures 6F,G**).

### Caffeine and theanine biosynthesis in tea flowers

**Figure 7:**
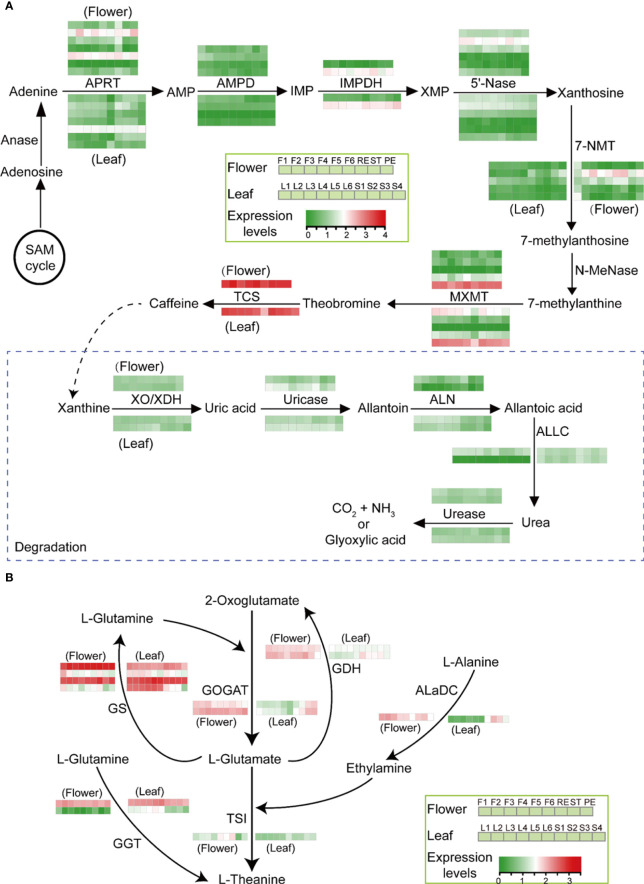
Caffeine and theanine biosynthesis in tea flowers. **(A, B)** Main biosynthetic route toward caffeine biosynthesis and degradation **(A)** and theanine biosynthesis **(B)** in tea developing flowers and leaves. AMP, adenosine monophosphate; IMP, inosine monophosphate; XMP, xanthosine monophosphate; Anase, adenosine nucleosidase; APRT, adenine phosphoribosyltransferase; AMPD, AMP deaminase; IMPDH, IMP dehydrogenase; 5′-Nase, 5′-nucleotidase; 7-NMT, 7-methylxanthosine synthase; N-MeNase, N-methylnucleotidase; MXMT, theobromine synthase; TCS, tea caffeine synthase; XO, xanthine oxidase; XDH, xanthine dehydrogenase; ALN, allantoinase; ALLC, allantoicase; GS, glutamine synthetase; GOGAT, glutamate synthase; GDH, glutamate dehydrogenase; AlaDC, alanine decarboxylase; GGT, γ-glutamyltranspeptidase; TSI, theanine synthetase. The log 10 (expression levels, TPM) of the genes is represented by a heat map.

Caffein and theanine are two other characteristic compounds in tea, and we analyzed their biosynthesis pathway in tea flowers. The gene expression analysis showed that the caffeine biosynthesis pathway is conservative in leaves and flowers. In both leaves and flowers, *tea caffeine synthase1* (*TCS1*) and *theobromine synthase* (*MXMT-5*) are the two major *methylxanthosine synthase* (*NMT*) genes with the highest expression levels (**Figure 7A**). Caffeine was highly accumulated in RE and ST compared with PE in flowers. However, those *NMT* genes all showed high expression levels, and no expression difference of those *NMT* genes was detected in PE, RE, and ST (**Figure 7A**).

Tea flowers also accumulated a large amount of amino acids, such as theanine and glutamate. Usually, theanine is synthesized in tea roots. In order to uncover the origin of theanine in flowers, the theanine biosynthesis pathway in flowers was analyzed. The gene expression analysis showed that *theanine synthetase* (*TSI*) and *alanine decarboxylase* (*AlaDC*), two theanine biosynthetic genes, were highly expressed in flowers compared with leaves as well as the genes involved in GS-GOGAT cycle (**Figure 7B**), suggesting the ability to synthesize theanine locally in tea flowers.

## Discussion

Tea flowers contain abundant metabolites, such as flavonoids, purine alkaloids, amino acids, and particularly volatiles, which were characteristic compounds of tea contributing to tea flavor formation (Zhao et al., 2020). Remarkably, tea flowers also contained much tea polysaccharide and saponin, which have been identified to contribute in improving the human immune system. Pharmacological experiments also showed that the extract of tea flowers did not show toxicity to normal cells (Li et al., 2011). Thus, tea flowers have tremendous potential for the development of new tea products such as tea flower drink and tea flower functional food.

Volatiles play critical roles in flavor formation for many drinks, such as the tea drink. The resource of tea volatiles is complex, including biosynthesis pathway in fresh leaves, such as terpenoid biosynthesis, and tea processing, such as lipid peroxidation products (Feng et al., 2019). In most plants, including tea, the flowers could synthesize, accumulate, and release abundant volatiles in native condition. Those components with unique aroma, particularly the terpenoid compounds, participated in attracting pollinators or defending the plant from diseases (Borg-Karlson et al., 2003; Parachnowitsch et al., 2012). Linalool is the predominant terpenoid volatile in tea flowers, as well as in leaves, which is catalyzed by monoterpene synthase *via* the conservative chloroplatinic MEP pathway (Liu et al., 2017). Our transcriptome data showed that no significant difference of expression levels of early genes involved in the MEP pathway was detected between tea leaves and flowers. This suggested that the divergent terpenoid compounds between tea leaves and flowers were determined by the divergent expression pattern of later genes, such as the *TPS* genes. In the tea genome, a rapid expansion of the *TPS* gene family was detected, leading to the high quality of tea (Xia et al., 2020). We found that only a small amount of *TPS* genes was selected synchronously to be expressed highly in leaves and flowers. Most *TPS* genes showed a significant expression difference between leaves and flowers, which also suggested that the function or expression pattern of the *TPS* gene family has been divergent during the tea plant evolution. The gene expression analysis also showed that, in tea flowers, the *TPS* genes involved in volatile terpene biosynthesis tended to be expressed in PE and ST and lowly in RE (**Figure 4**). However, the *TPS* genes involved in the biosynthesis of nonvolatile terpenes, such as saponins, were highly expressed in RE and PE and lowly in ST (**Supplementary Table S3**). In tea leaves, most *TPS* genes belonged to inducible-type genes and showed a low expression level in native condition (Xia et al., 2020). The accumulative expression of the *TPS* gene family further showed that the *TPS* gene family is highly expressed in flowers, particularly PE and ST, compared with leaves and stems, resulting in abundant volatiles in flowers.

Flavonoids, particularly flavonol and catechins, are the major characteristic compounds together contributing to the bitter flavor in tea drink (Zhao et al., 2020). The biosynthesis pathway of flavonoid in tea plants has been well characterized, which is derived from the phenylpropanoid pathway. In plants, the early genes involved in flavonoid biosynthesis are usually presented as gene families, such as *PAL* and *CHS* gene families. However, the copy number of the later genes involved in flavonoid biosynthesis was low—for example, both *ANS* and *ANR* only have two copies in the tea plant genome. The metabolic analysis showed that the leaves and flowers have a similar flavonoid profile. Interestingly, the transcriptome data revealed that different members of those in the early gene family showed a dramatic expression difference in leaves and flowers—for example, the *PAL* gene family has at least five copies, and *PAL2* is highly expressed in young leaves and stems, consistent with the flavonoid profile (**Figure 5**). However, the expression of *PAL1* and *PAL4* increased as the leaf and stem developed, which suggests that they are specially involved in lignin biosynthesis (**Figure 5**). In tea flowers, *PAL3* is the predominant gene, and its expression level is about 80% of the accumulative expressions of the *PAL* gene family (**Figure 5**).

CHS is the first limited enzyme in the flavonoid biosynthesis pathway. We also found that the members of the *CHS* gene family showed a significant expression preference in tea leaves and flowers. In tea leaves and stems, only four *CHS* genes were expressed; however, in tea flowers, six *CHS* genes were expressed, in which *CHS8* and *CHS9* were specially expressed in ST (**Figure 5**). Previous studies have shown that, in many plants, CHS proteins interacted with the upstream and downstream flavonoid biosynthetic enzyme proteins, forming super complexes (Jorgensen et al., 2005; Dastmalchi et al., 2016). Expectedly, we found some *FLS* genes, such as *FLS2*, specially expressed in ST. FLS2 could interact with CHS7 and CHS8, forming a complex to catalyze flavonol biosynthesis specially in flowers. Another evidence was that ectopically expressing the tea flower-specific *FLS* gene could not promote flavonol biosynthesis in tobacco leaves (Shi et al., 2021). Flavonol compounds were known as key components in plant fertility. In *Arabidopsis*, the absence of flavonols led to stamen defects (Zhang et al., 2021). In tea flowers, we also found that tea flavonol compounds were specially highly accumulated in PE and ST, and a set of biosynthetic genes, such as *CHS* and *FLS*, was specially highly expressed in PE and ST. It is observed that flavonol highly accumulated in stamens and play crucial roles in plant fertility. In tea leaves and flowers, a set of genes, not just one gene, in the whole pathway was synergistically selected for expression to synthesize divergent target compounds.

In tea flowers and leaves, the profile of flavonols was similar; however, the related biosynthetic genes were significantly different. The difference of those gene expressions was determined by upstream transcription factors. CsMYB55 was the major MYB transcription factor regulating flavonol biosynthesis in tea leaves (Zhao et al., 2021; Li et al., 2022b), but we did not detect the transcripts of *CsMYB55* in flowers. The regulatory mechanism of flavonol biosynthesis is entirely different in leaves and flowers. In the flowers of *Arabidopsis* and *Freesia hybrida*, the flavonol biosynthesis was redundantly regulated by another R2R3-MYB subgroup transcription factors MYB21 and MYB24 (Shan et al., 2020; Zhang et al., 2021). We also found that their homologs, *MYB148* and *MYB193*, were highly expressed in tea flowers, particularly in PE and ST, which indicates the conservative regulatory mechanism of flavonol biosynthesis in plant flowers. In *Arabidopsis* and *Freesia hybrida*, MYB21 and MYB24 could also regulate terpenoid biosynthesis in flowers (Yang et al., 2020). Tea plant flowers contained abundant terpenoid compounds, and MYB148 and MYB193 may be also involved in terpenoid biosynthesis regulation, and it is worthy to further explore it.

Catechins play important roles in tea flavor formation. Here we found in tea flowers, as well as tea leaves, that EGCG and ECG are the dominant catechins. The catechins mainly distributed in RE and ST. Catechins, particularly ECG, were perhaps advantageous to pollinators, such as bees, to improve the fertility rate (Gong et al., 2021). Catechin biosynthesis has been well studied in tea plants. The expression of many later genes involved in catechin biosynthesis showed a similar expression pattern in tea leaves and flowers. In tea plants, at least 10 MYB transcription factors regulated catechin biosynthesis together with WDR type TF TRANSPARENT TESTA GLABRA1 (TTG1) and bHLH type TF TRANSPARENT TESTA8 (TT8), in which the MYB184 has been identified as the key regulator regulating EGCG and ECG biosynthesis in tea leaves (Li et al., 2022a; Li et al., 2022c). The correlation analysis showed that the expression of *MYB184* and other MYB TFs had a significant correlation with the expression of *ANR* and *LAR*, two key catechin biosynthetic genes both in developing tea leaves and developing flowers, which indicated that catechin biosynthesis regulation is conservative in different tissues.

Caffeine not only has a wide range of pharmacological effects on the human body but also plays crucial roles in allelopathy, resistance to herbivore attacks, and pathogen infections (Brunyé et al., 2010; Mohanpuria & Yadav, 2010; Wright et al., 2013; Wang et al., 2016; Bojar et al., 2018). Interestingly, in flowers of tea, coffee, and citrus plants, an abundant level of caffeine has been detected (Huang et al., 2016). Caffeine was regarded as an agonist to improve the pollinator’s brain behavior (Wright et al., 2013). As expected, we found that caffeine mainly accumulated in ST. In tea leaves and flowers, *TCS1* and *MXMT-5* are the two dominant *NMT* genes, and the caffeine biosynthesis pathway is also conservative in leaves and flowers. However, the gene expression analysis showed that the *TCS1* and *MXMT* genes were highly expressed during flower development and in different parts, indicating that those *NMT* genes may be involved in other life processes.

Theanine is a nitrogen reservoir in tea roots and plays a critical role in nitrogen cycle in tea plants (Zhao et al., 2020). Theanine mainly was synthesized in tea roots and then transferred to other tissues, such as stems and leaves. Theanine contributes not only to the sweet and umami flavors of tea infusions but also to numerous health benefits on the human body. However, the physical functions of theanine on tea plants were not well uncovered. Tea flowers also contained abundant theanine and other amino acids, such as serine, proline, and isoleucine. Those amino acids may provide nutrition for flowering. A previous study also showed that theanine was synthesized in tea leaves (She et al., 2022). Here we also found that many genes involved in theanine biosynthesis, such as *TSI* and *AlaDC*, showed higher expression levels in tea flowers than in leaves. The gene expression analysis indicated that tea flowers may have the capacity of theanine biosynthesis locally, and the function of theanine in tea flowers also needs to be explored further.

Due to the few studies on the characteristics and physiological functions of metabolites in tea flowers, the comprehensive utilization of tea plant flowers is still few. Tea flowers contained abundant bioactivate compounds, such as tea polysaccharide, saponins, terpenoids, and flavonoids. Tea flowers have been an ideal natural resource of tea polysaccharide and saponins, and those compounds also showed a wonderful effect in improving the immune system. Here, based on target and non-target metabolic analyses, we also detected abundant metabolites in tea flowers, and the contents of many compounds in tea flowers are significantly higher than in tea leaves, particularly phenolic acids, such as GA. For those bioactive compounds, the tea flowers have been not only an important source of plant medicine but also has the potential to be developed into food and drink production. This study also used tea flowers as an addition to greatly improve the flavor of yellow large-leaf tea.

In conclusion, we integrated targeted and non-targeted metabolomic and transcriptomic analyses of developmental flowers and different parts of flowers to illustrate the biosynthesis and regulation mechanisms of volatile and non-volatile compounds by comparison with developing leaves and stems (**Supplementary Figure S6**). In addition, based on the abundant specific metabolites in tea flowers, we developed a new kind of application in tea processing. The study provides new insights into understanding the metabolism and transcriptome characteristics and nutritional value of tea flowers. It lays a foundation for future study to improve the comprehensive application of tea flowers.

## Data availability statement

The datasets presented in this study can be found in online repositories. The names of the repository/repositories and accession number(s) can be found in the article/**Supplementary Material**.

## Author contributions

PL conceived and designed the experiments. DT, YS, RY, JD, ZY, YL, WZ, and YY performed the experiments. FL, DT and YS analyzed the RNA-seq and metabolic data. PL, HW, LC, and JZ wrote the manuscript. All authors contributed to the article and approved the submitted version.

## Funding

This work is supported by the National Natural Science Foundation of China (32002089), the Anhui Provincial Natural Science Foundation (201902a05020408), and the College Students’ Innovative Entrepreneurial Training Plan Program (S202110364013).

## Acknowledgments

We thank Dr. Wei Tong for his assistance on bioinformatics analysis and manuscript discussion. The authors also thank the members in Prof. Zhao’s lab for all assistance with the experiments and data analyses.

## Conflict of interest

The authors declare that the research was conducted in the absence of any commercial or financial relationships that could be construed as a potential conflict of interest.

## Publisher’s note

All claims expressed in this article are solely those of the authors and do not necessarily represent those of their affiliated organizations, or those of the publisher, the editors and the reviewers. Any product that may be evaluated in this article, or claim that may be made by its manufacturer, is not guaranteed or endorsed by the publisher.

## Supplementary material

Supplementary Table 1 Metabolites identified by nontarget metabolic analysis in tea flowers and leaves.

Supplementary Table 2 Different expression genes in developing flowers.

Supplementary Table 3 Expression levels of key genes involved in flavonoid, caffeine, and terpenoid biosynthesis.

Supplementary Table 4 Expression levels of *TPS* gene family in tea flowers and leaves.
