# Supplementary material for: Integrating metabolite and transcriptome analysis revealed the different mechanisms of characteristic compound biosynthesis and transcriptional regulation in tea flowers
Source: Front Plant Sci. 2022 Sep 29;13:1016692. doi: 10.3389/fpls.2022.1016692 (PMC9557745; doi:10.3389/fpls.2022.1016692)
Supplement: Supplementary file 2 [file DataSheet_1.pdf]

Figure S1

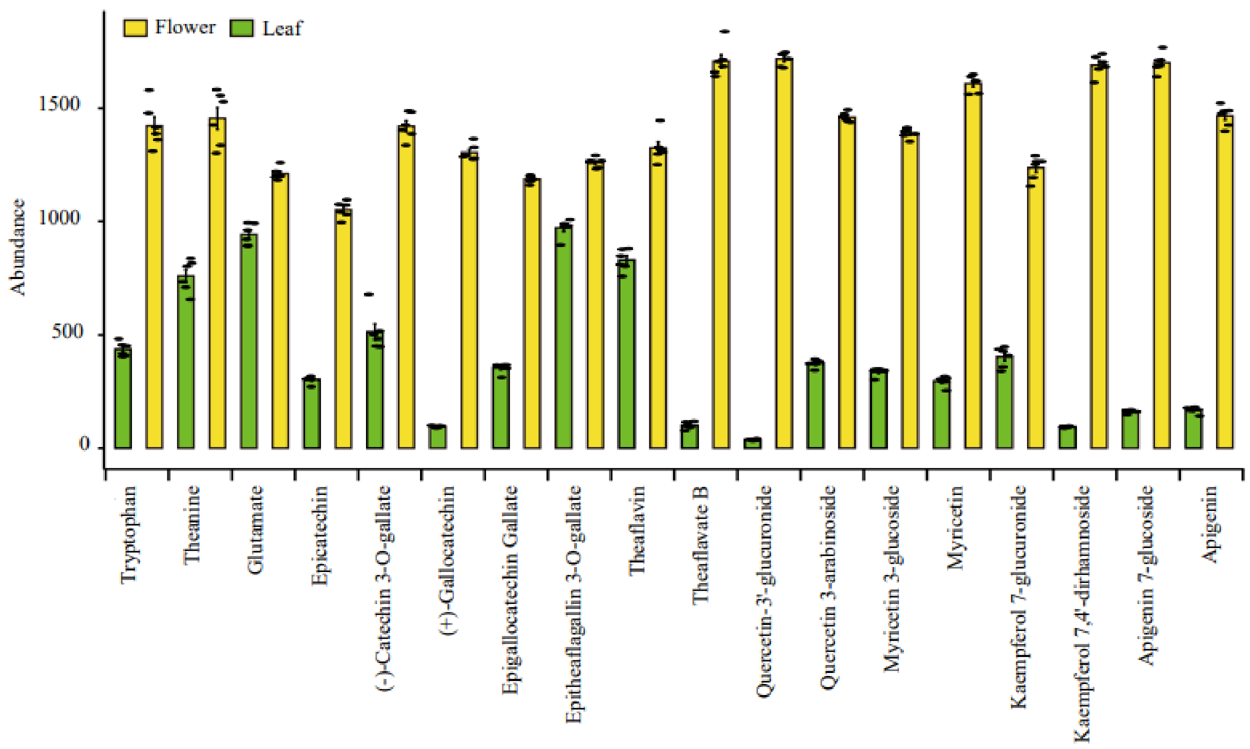

Figure S1. Differences in key metabolites between tea flowers and leaves.

Figure S2

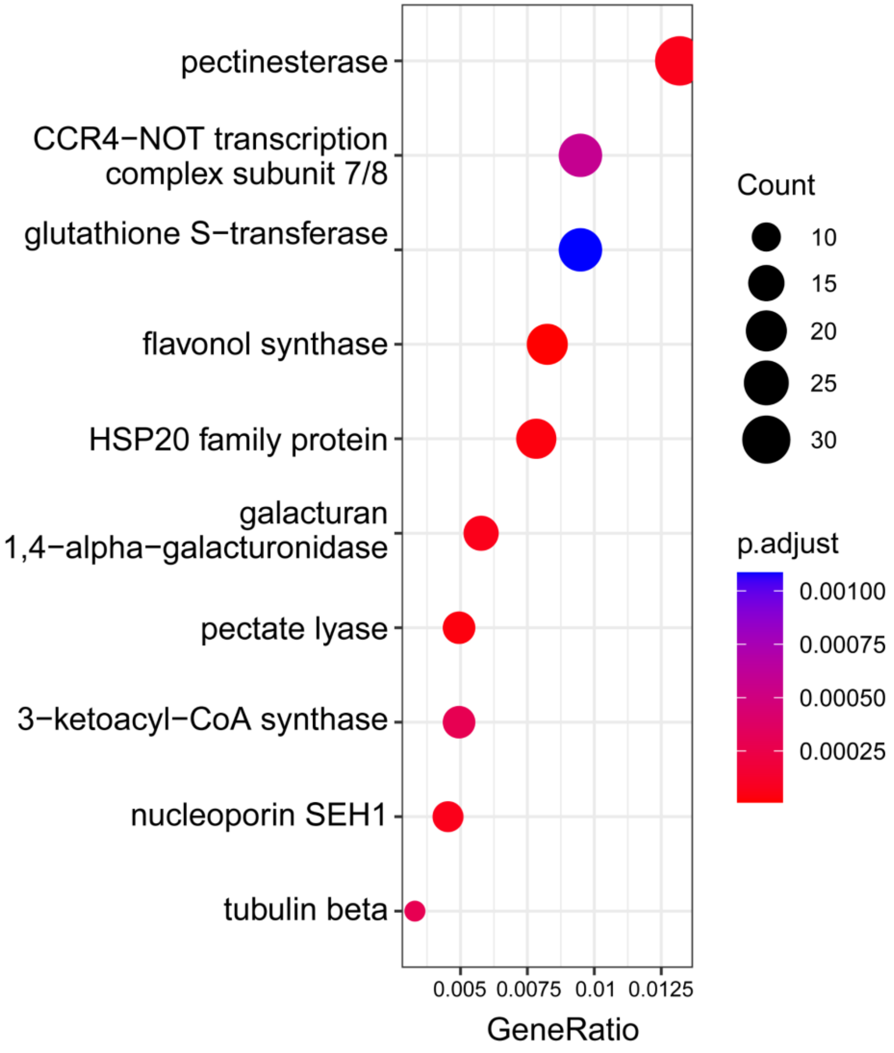

Figure S2. KEGG pathway analysis of different expression genes in developing tea flowers.

Figure S3

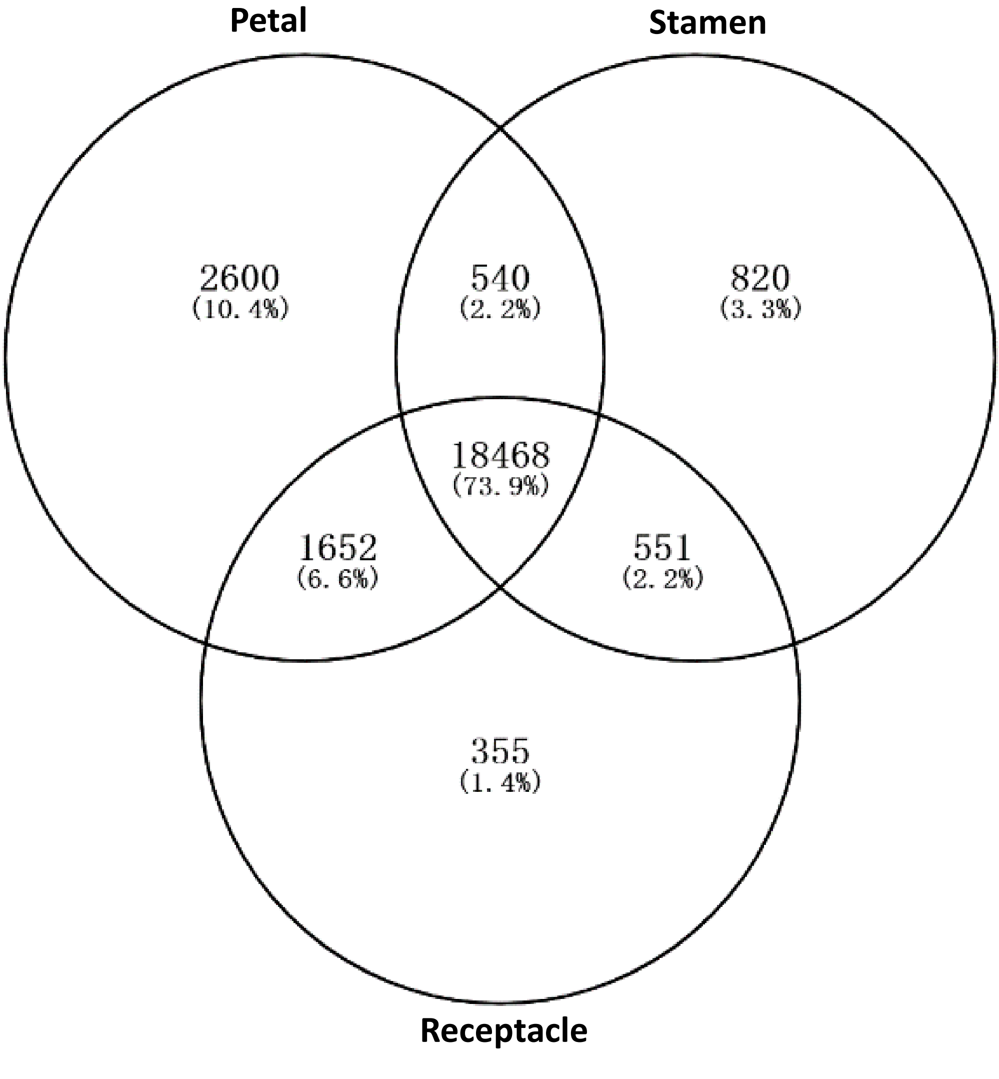

Figure S3. Venn showing the number of expression genes in petal, stamen and receptacle.

Figure S4

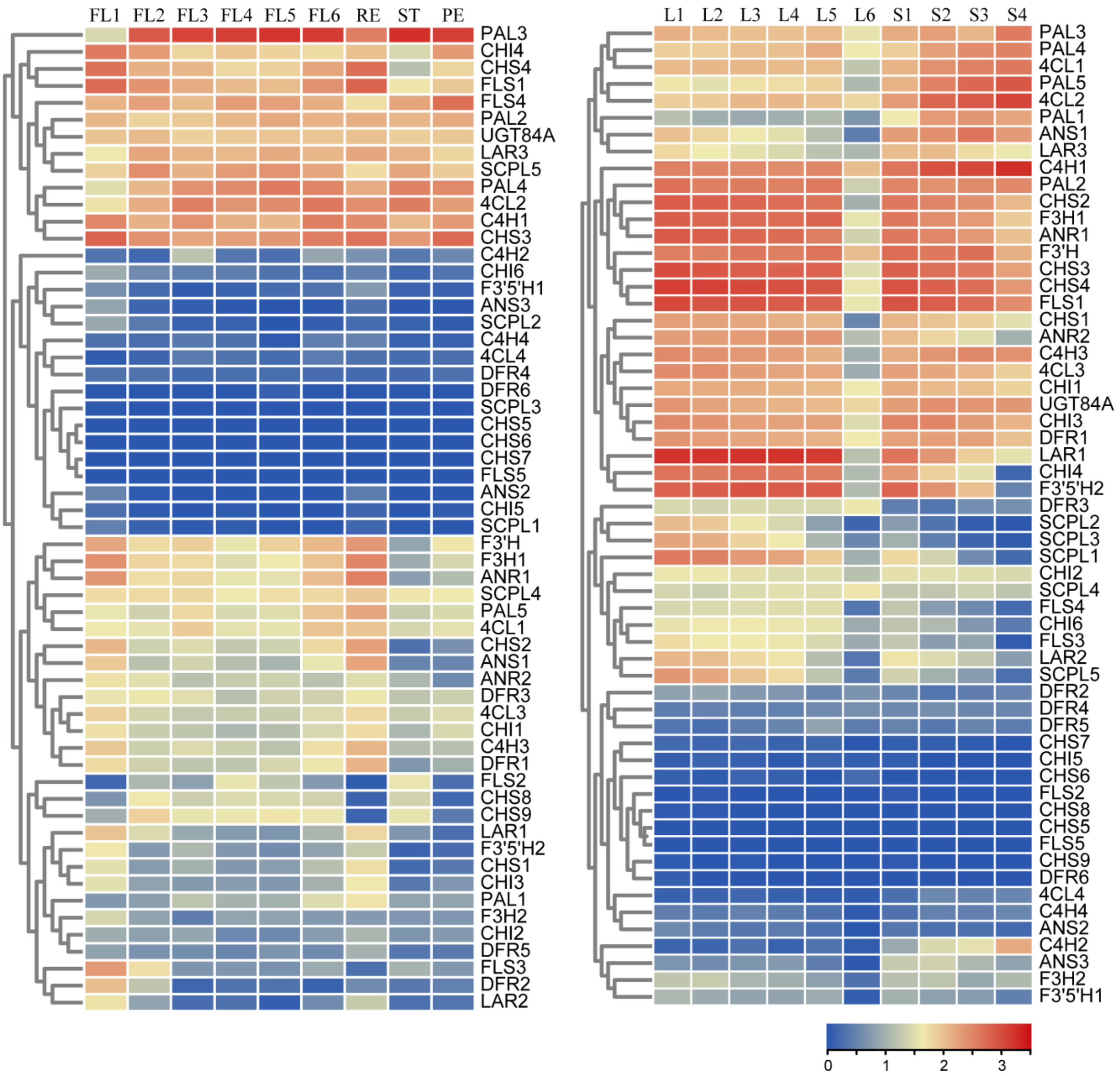

Figure S4. Heat map expression analysis of genes involved in flavonoid biosynthesis in tea flowers and leaves. The expression level (log10(FPKM)) of each gene is shown in the heatmap boxes.

Figure S5

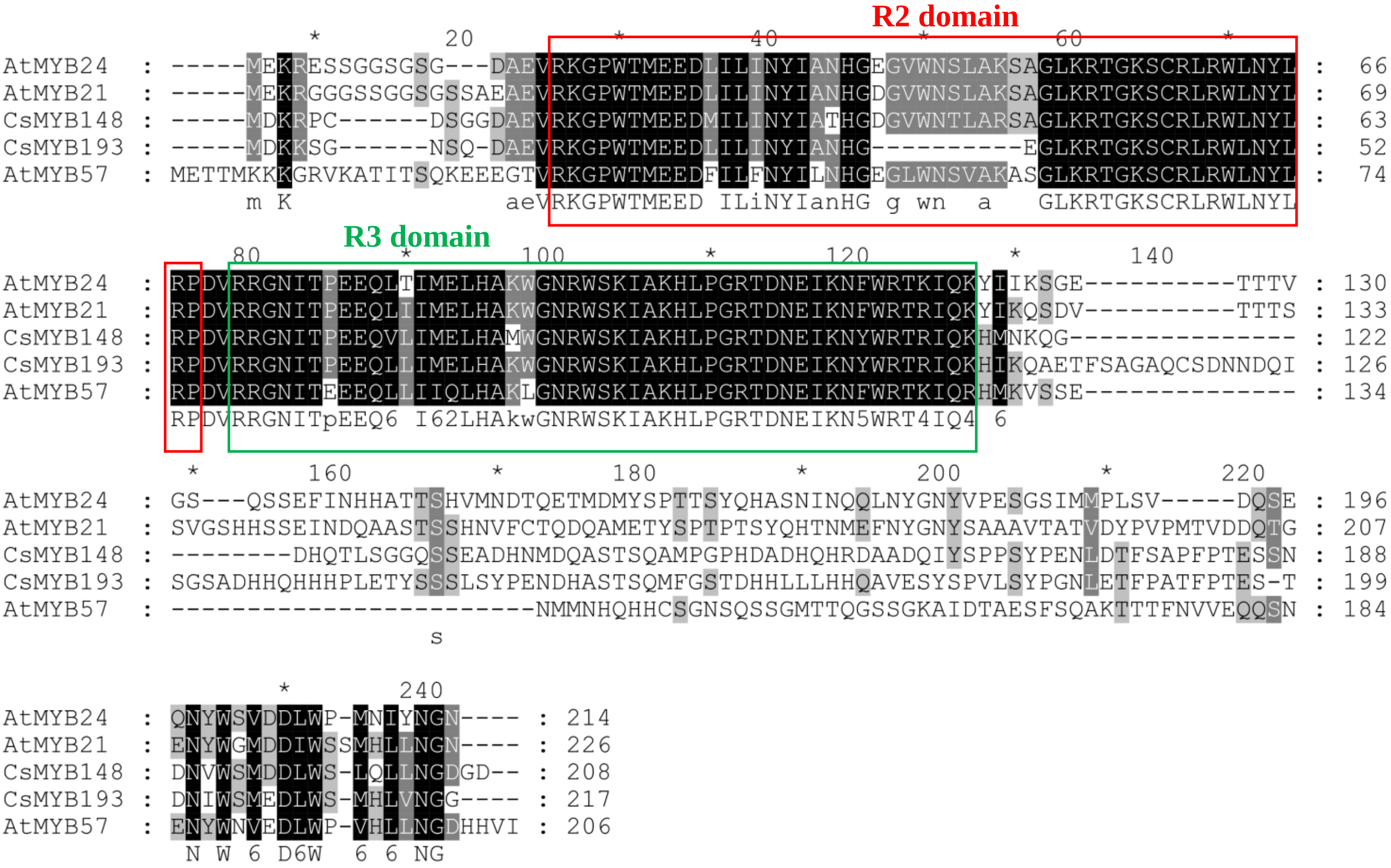

Figure S5. Alignment of tea MYB148 and MYB193 and related MYBs involved in regulating the flavonol biosynthesis in flowers. The R2R3 domain of those MYB proteins were remarked by rectangles.

Figure S6

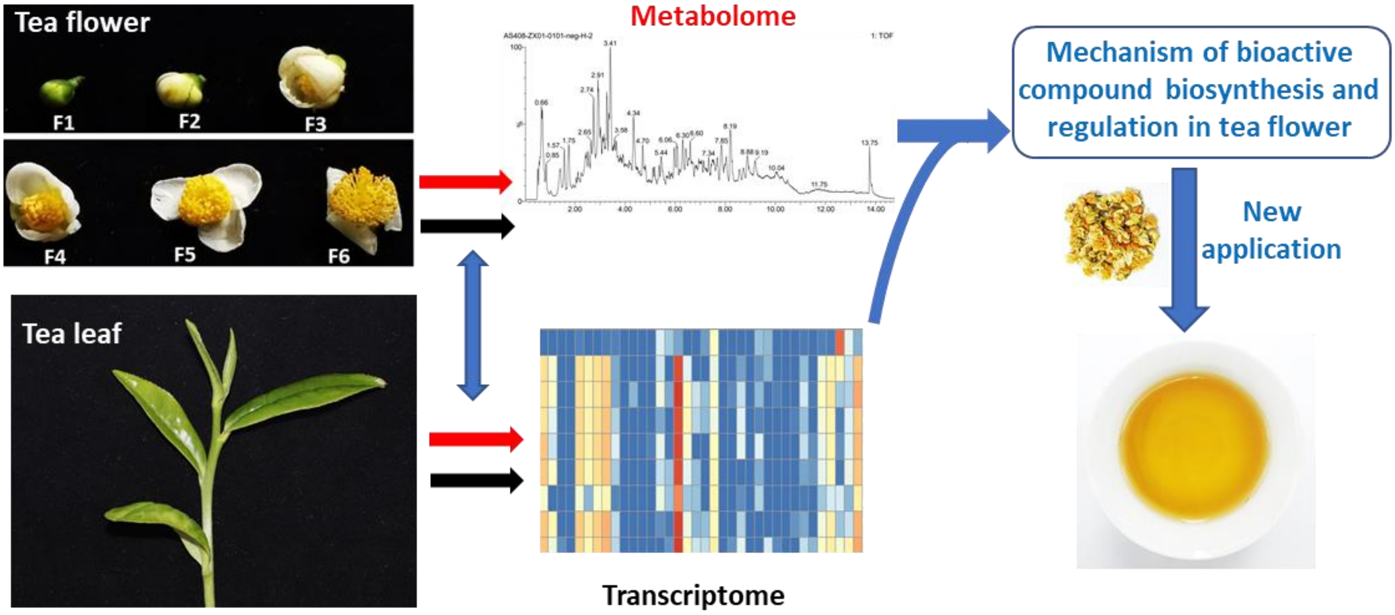

Figure S6. A summary of this study on tea flowers.
